# Severe Neonatal Hereditary Spherocytosis Presenting With Hyperbilirubinemia and Profound Anemia: A Case Report

**DOI:** 10.7759/cureus.117743

**Published:** 2026-10-10

**Authors:** Fatima Mbarki, Safae Rahmouni, Driss Oukachou, Skalli Khadija, Fouzia Hmami

**Affiliations:** 1 Department of Neonatology and Neonatal Intensive Care, Hassan II University Hospital, Faculty of Medicine and Pharmacy, Sidi Mohamed Ben Abdellah University, Fez, MAR

**Keywords:** eosin-5′-maleimide test, hereditary spherocytosis, neonatal hemolytic anemia, neonatal hyperbilirubinemia, neonate

## Abstract

Hereditary spherocytosis (HS) is an inherited erythrocyte membrane disorder with a variable clinical presentation during the neonatal period. Early unconjugated hyperbilirubinemia may be the first manifestation, whereas clinically significant anemia may develop later. A positive family history may therefore provide an important diagnostic clue. We report a term female neonate born at 38 weeks’ gestation to a mother with HS who had previously undergone splenectomy. Unconjugated hyperbilirubinemia developed on day 2 of life, with a total serum bilirubin level of 198.7 mg/L (339.6 µmol/L) and a negative direct antiglobulin test. Phototherapy was initiated; however, progressive hemolytic anemia subsequently developed, with hemoglobin decreasing from 16.0 g/dL on day 2 to 6.3 g/dL on day 30, accompanied by reticulocytosis (110 × 10⁹/L) and an elevated mean corpuscular hemoglobin concentration (36.8 g/dL). The eosin-5′-maleimide binding test demonstrated a 34.8% reduction in fluorescence, supporting the diagnosis of HS. Packed red blood cell transfusion corrected the severe anemia, with subsequent clinical improvement. This case highlights that neonatal HS may present with significant early hyperbilirubinemia followed by rapidly progressive severe anemia. In neonates with a family history of HS, early recognition and serial monitoring of bilirubin and hemoglobin levels are essential to guide timely management and reduce the risk of complications.

## Introduction

Hereditary spherocytosis (HS), also known as Minkowski-Chauffard disease, is the most common inherited red blood cell membrane disorder and an important cause of congenital hemolytic anemia. It results from quantitative or qualitative defects in proteins of the erythrocyte membrane skeleton, particularly ankyrin-1, band 3, α- and β-spectrin, and protein 4.2. These defects lead to progressive loss of membrane surface area, resulting in the formation of spherical, poorly deformable erythrocytes that are preferentially sequestered and destroyed in the spleen [1].

Inheritance is most often autosomal dominant, although autosomal recessive forms and de novo variants are recognized. HS occurs worldwide; prevalence estimates of approximately 1 in 2,000 to 1 in 5,000 individuals have been reported, with higher historical estimates in populations of Northern European ancestry [1,2].

The neonatal phenotype differs from the classic presentation seen later in childhood. Unconjugated hyperbilirubinemia is often the dominant early feature and may be severe, while anemia can be absent initially and become clinically significant during the following weeks. Splenomegaly is uncommon in neonates, and spherocytes may be sparse or absent on the initial peripheral blood smear. Consequently, the classic triad of jaundice, anemia, and splenomegaly is frequently incomplete at this age [1,3]. A known parental history of HS therefore provides a particularly valuable diagnostic clue.

We report a severe neonatal presentation of HS characterized by early unconjugated hyperbilirubinemia followed by rapidly progressive, profound hemolytic anemia in a term infant whose mother had established HS. This case highlights the importance of anticipatory surveillance in newborns with a positive family history and of extending follow-up beyond the initial control of jaundice.

## Case presentation

A female neonate was born at 38 weeks and three days of gestation to non-consanguineous parents. Her 28-year-old mother had been diagnosed with HS at the age of three years and had been followed since childhood. The maternal diagnosis was based on hematological findings, including a complete blood count, peripheral blood smear, and osmotic fragility testing. She underwent splenectomy in 2023. No genetic testing had been performed in the mother. The 33-year-old father had no relevant medical history. The pregnancy was regularly monitored and was complicated by gestational hypertension and gestational diabetes, with no clinical evidence of maternal-fetal infection. The infant was delivered vaginally, with a birth weight of 3,800 g and an uncomplicated postnatal adaptation.

Jaundice became clinically apparent on day 2 of life. Laboratory investigations revealed predominantly unconjugated hyperbilirubinemia, with a total bilirubin level of 198.7 mg/L (339.6 µmol/L), direct bilirubin of 4.8 mg/L, and indirect bilirubin of 193.9 mg/L (331.4 µmol/L), prompting initiation of phototherapy. Both the mother and infant were blood group O Rh-positive, and the direct antiglobulin test (DAT) was negative, making immune-mediated hemolysis unlikely (Table 1).

**Table 1:** Evolution of the main laboratory parameters Abbreviations: MCV, mean corpuscular volume; MCHC, mean corpuscular hemoglobin concentration; RDW, red cell distribution width.

Parameter	Day 2	Day 3	Day 4	Day 16	Day 30	Reference range
Hemoglobin (g/dL)	16.0	-	-	8.1	6.3	14–22 at birth
Hematocrit (%)	46	-	-	22.0	17.9	31–55 during the first month
MCV (fL)	104	-	-	95.5	93.8	85–115 during the first month
MCHC (g/dL)	35.0	-	-	36.8	35.2	30–36
RDW (%)	-	-	-	19.1	18.7	4–20 in neonates
Reticulocytes (×10⁹/L)	-	-	-	-	110	20–100 by 2–4 weeks
Indirect bilirubin (mg/L)	193.9	152	165	108	-	Interpret according to postnatal age in hours and neonatal bilirubin thresholds

Hyperbilirubinemia persisted, with total bilirubin levels of 161 mg/L on day 3 and 174 mg/L on day 4. Concurrently, progressive anemia developed, with hemoglobin decreasing from 16.0 g/dL on day 2 to 8.1 g/dL on day 16 and 6.3 g/dL on day 30. At day 30, the reticulocyte count was 110 × 10⁹/L. Erythrocyte morphology assessment reported anisochromia, without specific documentation of spherocytes. A representative peripheral blood smear image was not available. Given the severity and progression of the anemia, the infant was admitted to our neonatal intensive care unit for further investigation and management.

On admission at day 30, the infant was markedly pale and jaundiced but remained alert and reactive. Her body weight was 4,210 g. There was no hepatomegaly or splenomegaly, and the remainder of the physical examination was unremarkable.

The combination of early unconjugated hyperbilirubinemia, progressive severe anemia, and a negative DAT raised suspicion of a non-immune hemolytic disorder. Given the maternal history of HS, HS was considered the leading diagnosis. Because of the profound anemia, packed red blood cells were prepared for transfusion. Before transfusion, blood samples required for the etiological workup were obtained in a single sampling session to avoid repeated phlebotomy in the setting of severe anemia and to prevent transfusion-related interference with erythrocyte-based diagnostic testing. The workup included the eosin-5′-maleimide (EMA) binding test, glucose-6-phosphate dehydrogenase (G6PD) activity, and erythrocyte pyruvate kinase activity.

Following collection of the diagnostic samples, the infant received a red blood cell transfusion, which was well tolerated. A post-transfusion complete blood count showed an increase in hemoglobin from 6.3 to 11.0 g/dL, accompanied by clinical improvement. The infant was subsequently discharged in stable condition with scheduled outpatient follow-up.

The results of the etiological investigations became available during the subsequent follow-up visit. The EMA binding test performed by flow cytometry demonstrated a 34.8% reduction in erythrocyte fluorescence (laboratory reference value <16%), strongly supporting the diagnosis of HS. G6PD activity was 386, within the laboratory reference range of 291-411, while erythrocyte pyruvate kinase activity was elevated at 65.5 IU/g Hb (reference range, 7.6-28.6 IU/g Hb), thereby providing no evidence of pyruvate kinase deficiency. Serum ferritin was 148.3 ng/mL and thyroid-stimulating hormone was 3.55 µIU/mL. Taken together, the positive maternal history, early predominantly unconjugated hyperbilirubinemia, severe DAT-negative anemia, and abnormal EMA binding test supported the diagnosis of neonatal HS (Table 2).

**Table 2:** Etiologic and confirmatory investigations Abbreviations: EMA, eosin-5′-maleimide; G6PD, glucose-6-phosphate dehydrogenase; TSH, thyroid-stimulating hormone.

Test	Result	Reference range/interpretation
Direct antiglobulin test	Negative	Normal/expected: Negative
EMA binding test (flow cytometry)	Positive; 34.8% reduction in fluorescence	Normal: <16% reduction in fluorescence
G6PD activity	386 U/10¹² red blood cells	291–411 U/10¹² red blood cells
Erythrocyte pyruvate kinase	65.5 IU/g Hb	7.6–28.6 IU/g Hb
Ferritin	148.3 ng/mL	50–973 ng/mL (0–4 weeks)
TSH	3.55 µIU/mL	0.7–11.0 µIU/mL (6 days–2 months)

Approximately two weeks after transfusion, the infant was reassessed clinically. She remained clinically stable but appeared mildly pale. A repeat complete blood count revealed a decline in hemoglobin to 8.1 g/dL, indicating early recurrence of significant anemia after transfusion. Given the rapid decrease in hemoglobin, the infant was urgently referred for pediatric hematology consultation for further evaluation and management. Close hematologic follow-up with serial monitoring of hemoglobin, reticulocyte count, and markers of hemolysis was planned.

## Discussion

HS is an important cause of intrinsic red cell hemolysis in childhood, but recognition during the neonatal period can be challenging because the phenotype is often incomplete. Jaundice is the most frequent presenting feature, whereas more than half of affected neonates may not be anemic during the first week and splenomegaly is uncommon [1,3]. The present case followed a clinically important pattern: significant jaundice appeared first, while severe anemia evolved progressively over the subsequent weeks.

Early unconjugated hyperbilirubinemia in HS reflects increased bilirubin production from hemolysis and may reach clinically important levels. Recent neonatal reviews emphasize that HS should be considered among causes of early or significant hemolytic jaundice, particularly when the DAT is negative [1]. This is clinically relevant because hemolysis is a recognized bilirubin neurotoxicity risk factor, and contemporary guidance recommends treatment decisions based on gestational age, bilirubin concentration, age in hours, and neurotoxicity risk factors [4]. In our patient, jaundice developed on day 2 and required phototherapy, while the DAT was negative and maternal and neonatal blood groups were identical.

A notable feature of this observation was the delayed but profound fall in hemoglobin, from 16.0 g/dL on day 2 to 6.3 g/dL by day 30. Similar neonatal reports describe infants whose bilirubin abnormalities precede severe anemia requiring transfusion during the first weeks of life [4,5]. This temporal dissociation is clinically important: successful treatment of the initial jaundice does not eliminate the subsequent risk of symptomatic anemia. Serial hemoglobin and reticulocyte measurements should therefore continue after discharge in neonates with suspected or confirmed HS [3].

Family history substantially strengthened the diagnostic reasoning in this case. Approximately two-thirds of neonates with HS have an affected parent, and a history of chronic hemolytic anemia, recurrent jaundice, early gallstones, or splenectomy should be actively sought [3,5]. Our patient’s mother had childhood-onset HS and had undergone splenectomy, making inherited membranopathy a high-priority diagnosis once DAT-negative hemolysis became apparent. Nevertheless, a negative family history does not exclude HS because recessive inheritance and de novo variants occur [1,3,6].

Routine erythrocyte indices can provide useful early clues. Christensen and Henry reported that an MCHC ≥36.0 g/dL had 82% sensitivity and 98% specificity for identifying HS among neonates in their cohort [1]. More recent work also highlights elevated MCHC, increased RDW, and the MCHC/MCV ratio (HS index) as useful screening signals, although none should be used in isolation [1,6]. In our patient, MCHC reached 36.8 g/dL on day 16, and RDW was elevated, supporting the suspicion of a membrane disorder in the appropriate clinical context.

Peripheral blood morphology remains informative but is less reliable in neonates than in older children. Spherocytes may be few or absent, and the reticulocyte response can appear modest relative to the degree of anemia [1,3,5]. Furthermore, spherocytes are not specific to HS and may also occur in immune hemolysis. Accordingly, diagnosis should integrate the clinical phenotype, family history, DAT, erythrocyte indices, morphology, and confirmatory testing rather than rely on a single finding.

The EMA binding assay is now widely used as a confirmatory test for HS. By flow cytometry, reduced EMA fluorescence reflects abnormalities involving erythrocyte membrane proteins, particularly band 3-associated complexes. Current diagnostic reviews place EMA testing among the most useful specialized assays, alongside osmotic fragility-based methods and, when required, molecular testing [2-4,6]. In this infant, a 34.8% reduction in fluorescence, together with the characteristic clinical and familial context, provided strong confirmation of HS.

The principal neonatal management priorities are control of hyperbilirubinemia and treatment of clinically significant anemia. Phototherapy should be initiated according to age-specific treatment thresholds, with escalation of care and exchange transfusion when indicated by bilirubin level and neurotoxicity risk [4]. Severe or symptomatic anemia may require a red blood cell transfusion [2,3]. Our patient required phototherapy during the early neonatal period and later red blood cell transfusion when hemoglobin reached 6.3 g/dL, followed by clinical and hematologic improvement.

This case reinforces the need for anticipatory care when HS is known in a parent. Surveillance should not end once bilirubin concentrations fall; hemoglobin, reticulocyte count, and clinical signs of anemia should be reassessed during the first weeks of life. Early recognition also permits counseling regarding later hemolytic or aplastic episodes and longer-term complications of HS [3,6-8].

## Conclusions

Neonatal HS may present as early unconjugated hyperbilirubinemia followed by rapidly progressive, profound hemolytic anemia. A positive parental history should prompt targeted evaluation and planned postnatal surveillance even when the infant is initially clinically well. In a DAT-negative neonate with evidence of hemolysis, elevated erythrocyte indices and EMA binding analysis can support timely diagnosis. Continued hematologic follow-up after resolution of jaundice is essential because severe anemia may emerge during the first weeks of life.
